# Report Relative to the Epidemic of Upper Languedoc

**Published:** 1783

**Authors:** 


VII. *Rapport ſur l'Epidemie du Haut Languedoc, lû a la Société Royale de Medecine le* 19 *Juillet* 1782. i. e. *Report relative to the Epidemic of Upper Languedoc, read at the Royal Medical Society July* 19, 1782. 4to. Paris, 1782.

IN the month of September 1781, an epidemic fever began to prevail at Caſtelnaudary, and ſpread from thence over the greateſt part of Upper Languedoc. The French phyſicians call it *la ſuette miliaire.* It is ſaid to have been characterized by the copiouſneſs and long continuance of the ſweats. In a few days after the attack an eruption appeared, which in ſome patients reſembled ſcarlatina, in others eryſipelas, and was almoſt always accompanied with miliary puſtules, and ſometimes with petechiæ.

The fever commonly laſted about ſeven days, and there was uſually an exacerbation of all the ſymptoms every twenty-four hours. The ſweating took place from the attack, and continued during the whole of the diſeaſe. The eruption generally generally appeared about the third day, and continued till the ſeventh, when a freſh eruption appeared, which terminated the diſeaſe, and was followed by a complete deſquamation of the cuticle.

The Univerſity of Montpellier having been conſulted, gave it as their opinion, that it was a diſeaſe ſimilar to the ſweating miliary fever (*ſuette miliaire*) that prevailed at Guiſe in Normandy in 1759, and to the ſweating fever (*ſuette*) of Picardy and Normandy, a fever which is by Dr. Cullen, with good reaſon, conſidered as a ſpecies of typhus.

This diſeaſe has given riſe to ſeveral pamphlets, the titles of which will be found in the quarterly catalogue of our preſent number. In almoſt all of them the rules of treatment preſcribed were to encourage the ſweating and eruption by a heating regimen. The fever, in conſequence of this treatment, became every day more and more fatal, and having at length reached Toulouſe, ſpread an univerſal conſternation. Letters and memoirs came in from all parts of the province to the Royal Medical Society, and a new plan of cure was recommended, the reſult of which is deſcribed in the work before us. The new method conſiſted in taking away blood at the beginning beginning when inflammatory ſymptoms were preſent, in expoſing the patients freely to the cold air, and in preſcribing a cooling regimen. This was adopted univerſally, and with the beſt ſucceſs.

M. Brunet, phyſician at Toulouſe, went to Sarlat, where there were 600 perſons labouring under this fever, and where 41 had died. He immediately cauſed the above mode of treatment to be adopted, and after that there died only one patient, and this was a woman whom her huſband had obſtinately refuſed to ſubmit to the new method. All the others were cured without venæſection, ſimply by a cool regimen and expoſure to the air. M. Pujol, correſpondent of the Society at Caſtres, in a letter written ſoon after this to the Society, obſerves, " Since the " charm is deſtroyed, and the eyes of the public " are opened, we are become wiſe by our miſ- " fortunes. Since this happy revolution, the " epidemical diſeaſe, which had made ſo much " noiſe and occaſioned ſuch alarm, has loſt, " even in the minds of the public, all its im- " portance."

One patient, we are told, who was held down in his bed by four men, and who in his delirium tried to diſengage himſelf, was found covered with with bed-cloaths, the curtains of the bed being at the ſame time drawn cloſe, and the windows and doors of the room kept ſhut, with a great fire. He had ſweated much the firſt day, but the ſweating having ſtopped, his ſkin became parched with heat, and he was ſeized with violent delirium. A phyſician who found him in this ſtate, opened the doors and windows of the apartment, put out the fire, and placed the patient upon the floor, in his ſhirt, cloſe to the window. In a quarter of an hour the delirium went off, the patient ſeemed diſpoſed to ſleep, and ſoon recovered.

The Society give a ſuccinct view of ſimilar fevers as they have prevailed at different times in different provinces of France. In all of theſe the utility of the antiphlogiſtic plan of treatment, and the pernicious effects of a contrary method, were clearly aſcertained.

